# Supplementary material for: An open-source framework for large-scale, flexible evaluation of biomedical text mining systems
Source: J Biomed Discov Collab. 2008 Jan 29;3:1. doi: 10.1186/1747-5333-3-1 (PMC2276192; doi:10.1186/1747-5333-3-1)
Supplement: Additional file 1 — Evaluation platform source code. The additional file contains the software evaluation framework discussed in this paper. The most current release can be downloaded from SourceForge.net [1]. See the accompanying README file for instructions on installation and use. Also included in the distribution is a collection of UIMA wrappers for some commonly used BioNLP tools and annotated corpora. [file 1747-5333-3-1-S1.zip › bionlp_uima_repository-1.0.1/docs/api/edu/uchsc/ccp/util/external/abgene/AbGeneEISystem.html]

AbGeneEISystem (Center for Computational Pharmacology Util/UIMA API)


|  |  |  |
| --- | --- | --- |
| **Overview** \| **Package** \| **Class** \| **Tree** \| **Deprecated** \| **Index** \| **Help** | |  |
| PREV CLASS   NEXT CLASS | **FRAMES**    **NO FRAMES**     **All Classes** |  |
| SUMMARY: NESTED \| FIELD \| CONSTR \| METHOD | DETAIL: FIELD \| CONSTR \| METHOD |  |


---


## edu.uchsc.ccp.util.external.abgene Class AbGeneEISystem

```
java.lang.Object
  edu.uchsc.ccp.util.external.abgene.AbGeneEISystem
```

**All Implemented Interfaces:**: EIFileBasedSystem, EISystem

---

``` public class AbGeneEISystem extends Object implements EIFileBasedSystem ```

**Author:**
:   williamb
    NOTE: this will only run in a LINUX/UNIX environment due to LINUX specific commands in KeXEISystem.createExecFile

---

**Constructor Summary**

`AbGeneEISystem(String baseDirectory)`
  
          Initialize a new AbGene EI System


| **Method Summary** | |
| --- | --- |
| `List<TextAnnotation>` | `getEntitiesFromFile(String inputFile)`             Works as it would when using AbGene from the command line. |
| `List<TextAnnotation>` | `getEntitiesFromString(String inputText, String inputDocumentID)`             Take a String as input, create a temporary input file, then run AbGene just as you would from the command line. |
| `String` | `getOutputFilename()`             Return the outputFileName and path |

**Methods inherited from class java.lang.Object**

`clone, equals, finalize, getClass, hashCode, notify, notifyAll, toString, wait, wait, wait`

**Constructor Detail**

### AbGeneEISystem

```
public AbGeneEISystem(String baseDirectory)
```

:   Initialize a new AbGene EI System

    **Parameters:**: `baseDirectory` - - the absolute path to the directory where the KeX exprot function is located - there MUST exist a baseDirectory/work directory


**Method Detail**

### getOutputFilename

```
public String getOutputFilename()
```

:   Return the outputFileName and path

    :   **Specified by:**: `getOutputFilename` in interface `EIFileBasedSystem`
    :   **Returns:**: - String containing the name of the file where the system's output is stored

---


### getEntitiesFromFile

```
public List<TextAnnotation> getEntitiesFromFile(String inputFile)
```

:   Works as it would when using AbGene from the command line. The input argument is a file where each line represents an individual
    document to be tagged with gene names. Each line must take the form of:  

    :   **Specified by:**: `getEntitiesFromFile` in interface `EIFileBasedSystem`
    :   **Returns:**: - ArrayList of TextAnnotations

---


### getEntitiesFromString

```
public List<TextAnnotation> getEntitiesFromString(String inputText,
                                                  String inputDocumentID)
```

:   Take a String as input, create a temporary input file, then run AbGene just as you would from the command line. The document ID is
    appended to the input string in order to create the expected input format.

    :   **Specified by:**: `getEntitiesFromString` in interface `EISystem`


---


|  |  |  |
| --- | --- | --- |
| **Overview** \| **Package** \| **Class** \| **Tree** \| **Deprecated** \| **Index** \| **Help** | |  |
| PREV CLASS   NEXT CLASS | **FRAMES**    **NO FRAMES**     **All Classes** |  |
| SUMMARY: NESTED \| FIELD \| CONSTR \| METHOD | DETAIL: FIELD \| CONSTR \| METHOD |  |


---
